# School EFL Teachers’ Research Identity Construction in the Chinese University–School Community

**DOI:** 10.3389/fpsyg.2022.897425

**Published:** 2022-06-23

**Authors:** Xuemei Wang, Yanhua He

**Affiliations:** School of English Studies, Shanghai International Studies University, Shanghai, China

**Keywords:** school EFL teacher, research identity, identity construction, University-School community, Activity Theory

## Abstract

Despite a relatively large number of studies on teachers’ identity development in the University-School community, few studies have explicitly focused on school EFL teachers’ research identity construction. This study adopts the Activity Theory and examines three English as a foreign language (EFL) teachers from three middle schools in a University–School community in China. It investigates how three teachers constructed their research identity and what factors influenced the construction of their identities within the University–School activity system from the dual perspectives of the school and university teachers. Data are collected through semi-structured narrative interviews, triangulated by documents such as meeting minutes, and then analyzed by NVivo 12. The findings of this study show that (1) the University–School collaborative program helps form a University–School community of both teaching and research; (2) in this community, school EFL teachers continuously construct their identities in a spiral process of “practitioner” and “researcher”; (3) it highlights the internal factors containing the research experience and the stage of career and the external factors including the curriculum reform context and the communication in the community. The findings carry important implications for school EFL teachers’ research identity construction and professional development in the University–School cooperation.

## Introduction

English as a foreign language (EFL) teachers’ professional identity construction is currently one of the most comprehensively researched topics in language teacher development (Johnson and Golombek, 2020). EFL teachers’ professional identity was formerly studied as a negotiation process in specific teaching contexts (He and Lin, 2013; Han, 2021; Jiang, 2022). There is an increasing tendency to study EFL identity development through academic research (Schutz and Hoffman, 2017; Yuan and Burns, 2017; Kenan and Demet, 2018). However, the study of EFL teachers’ research identity (i.e., professional identity as a researcher) has generally focused on university teachers (Hanks, 2014; Xu, 2014; Kenan and Demet, 2018; Jiang and Zhang, 2021). Such participant imbalance reflects the general scholarly neglect of EFL teachers’ research life (Zhuang and Qi, 2015; Yuan and Zhang, 2020). Thus, it is necessary to expand current research to EFL teachers in other types of schools.

School EFL teachers encounter challenges in constructing their professional identities in various contexts (Pennington and Richards, 2016). To address it, Bukor (2015) proposed that professional development programs, such as University–School (hereafter referred to as U-S) collaborations (Yuan, 2016), are conducive to constructing EFL teachers’ identities. However, very few studies on the U-S community deal with school teachers’ research identity construction (Yuan and Burns, 2017). Therefore, the current research seeks to examine EFL teachers’ research identity construction and the role of the U-S community in this process from a dialectic approach that integrates the perspectives of both the middle school teachers and their counterpart university teachers.

Most studies in the field focus on EFL teachers’ identity construction, and studies about school EFL teachers rarely intersect with those about university teachers. Although more school EFL teachers are involved in research in U-S communities, these communities mainly serve the purpose of cultivating pre-service teachers (i.e., student-teacher development programs) instead of addressing problems in teaching practices. Moreover, teachers’ research identity is not the focus of previous studies. By focusing on two activity systems in the U-S community, the present study attempts to address those lacunae via a case study of three school EFL teachers by exploring their research identity from the school and university teachers’ perspectives. According to the Sociocultural Theory (SCT), human behaviors are the joint results of socially and culturally constructed forms of mediation (Lantolf and Thorne, 2006). The current study thus posits that EFL teachers’ identity mediates and is mediated by relevant activity systems. Drawing on Activity Theory (Engeström, 1999), this study tries to bring about enlightenment from three aspects. First, it provides valuable insights into how the U-S community is constructed and how the U-S community mediates teachers’ identity. Then, it proposes the influencing factors that play a paramount role in school EFL teachers’ identity construction. Finally, it aims to develop school teachers’ research identity and improve collaboration in the U-S community.

## Literature Review

### EFL Teachers’ Professional Identity

Teachers’ identity has been defined as “a certain kind of person” (Gee, 2001: 99) in situated communities (Yuan and Burns, 2017). It is contextually located and could be mediated and remediated by the interaction within the professional contexts (Beijaard et al., 2004; Beauchamp and Thomas, 2009). Identity in different contexts develops in an ongoing and dynamic process involving interactions and struggles with internal and external factors (Vargehese et al., 2005). The consequent identity performance includes acceptance, reinforcement, weakening, or challenge (Pennington and Richards, 2016). There are three research foci regarding EFL teachers’ professional identity and its influencing factors. The first concerns such internal factors as stages of career development (Steffy, 1989; Xu, 2014; Pennington, 2015), attitude about research (Williams and Coles, 2007; Livingston, 2016), engagement in the community (Goodnough, 2010; Yuan and Burns, 2017), and emotion toward research (Li and Liu, 2021). The second concerns external factors related to identity, such as teaching reform (He and Lin, 2013; Aderet-German et al., 2019; Jiang, 2022). The last one focuses on its relationship with internal and external factors (Peker et al., 2020).

The above literature manifests two significant perspectives in teacher identity research. First, its insists that the teachers’ identity is mainly constructed from language teaching practice under the influence of both internal and external factors (Yuan, 2020). Second, it proposes that teaching and research both play essential roles in constructing a teacher’s identity (Pennington and Richards, 2016; Schutz and Hoffman, 2017).

### EFL Teachers’ Research Identity Construction

In line with the classification of teachers’ professional identity construction in their academic development (Gee, 2001; Pennington and Richards, 2016), EFL teachers’ research identity construction highlights the importance of research in identity development. Teachers devoted to research often seek journal publications, research projects, conference experiences, academic visits, and advanced studies for continuous reflection and academic development in their career (Flowerdew and Wang, 2015: 82; Zacher et al., 2019: 365; Liu et al., 2021). Given this shift of emphasis, most studies on EFL teachers’ research identity seek to theorize their practices *per se* or the process of how practices turn into knowledge, especially in community contexts (Pennington and Richards, 2016). Recently, the U-S community has been in place to promote teachers’ professional development (Burns, 2009; Hanks, 2014, 2019; Yuan and Burns, 2017). In terms of the U-S community, existing studies mainly focus on the research-oriented U-S community and the practices of U-S collaborative projects aiming to enhance the professional development of teachers and teacher educators (Yuan and Burns, 2017; Kenan and Demet, 2018), instead of focusing on the teaching problems in reforms (He and Lin, 2013).

Moreover, in terms of the function of the U-S community, some studies have shown that U-S communities could facilitate teachers’ identity construction. For example, some scholars uncovered the ongoing research identity development of how “practitioners” become “researchers” through action research with active engagement or exploration that sheds light on specific teaching practices (Allwright, 2003; Goodnough, 2010; Hanks, 2014, 2019; Yuan and Burns, 2017; Schutz and Hoffman, 2017: 9). Meanwhile, other research works have shown that community might constrain teachers’ identity formation, for power differentials might reduce their status to mere “participants” (Chan and Clarke, 2014) and cause tensions or conflicts in the community (He and Lin, 2013; Yan and Yang, 2017). In this light, teachers’ research identity exploration was sidelined regardless of its equal importance to teaching in teachers’ professional development (Zhuang and Qi, 2015; Hanks, 2019). Thus, the study on school EFL teachers’ research identity construction needs to be pinpointed. Adopting the Activity Theory (Engeström, 1999), this study seeks to explore how the U-S community is and how school EFL teachers’ research identity evolves or develops through the community.

## Theoretical Framework

By drawing on the Activity Theory (hereafter referred to as AT) initiated by Vygotsky (1978) and developed by Leont’ev (1981) and Engeström (1987, 1999), this study explores school EFL teachers’ research identity construction within the U-S community in China. Initially, Vygotsky (1978) constructed the basic triangle of the subject, object, and mediation artifact, indicating that learners are active participants instead of passive recipients of learning. Subsequently, Leont’ev (1981) partitioned this learning activity into three levels (activity, action, and operation) to further elaborate on the different actions toward the object and the correlation between action and activity. His theory was that activity might be conducted with different actions, and one action might result in different activities. Vygotsky and Leont’ev’s theorization of activity is regarded as the first generation of AT. To address the specific factors in the activity, Engeström (1987) conceptualized activity as the interaction between the subject and object, mediated by five components: community (i.e., the group that the subject belongs to), tool (i.e., means used in the activity), rules (i.e., regulations in the activity), division of labor (i.e., how tasks are assigned), and outcome (i.e., the end general result). This theorization constitutes the second generation of AT. However, real-world practices often intermingle multiple perspectives throughout the interaction among different activity systems. As such, Engeström (1999) further developed the third generation of AT in which the elements in the activity system 1 and those in the activity system 2 may overlap for a shared object in the joint activity system.

Therefore, according to the third-generation AT model, subjects from the university community and the school community participate in the activities of their respective systems but jointly foster a shared object in the U-S community. More specifically, the third-generation AT helps researchers explore the elements and functions of each system to identify the most influential element in school teachers’ identity construction. Researchers have addressed EFL teachers’ identity development within specific activity systems based on this theoretical rationale. For example, He and Lin (2013) studied a student EFL teacher’s identity formation within the U-S community in China. However, this study only focused on one student-teacher rather than in-service teachers. It also only explored the U-S community of a practicum project among university students other than a curriculum reform project jointly participated by middle school and university teachers. Based on the third-generation AT model, the current study surveyed three school EFL teachers from three different U-S communities in China. It investigated how these three teachers constructed their EFL teacher–researcher identity and explored the influencing factors in their identity construction within the U-S activity system. Finally, we aim to address three research questions:

1. How is the U-S community in the Chinese EFL curriculum reform constituted from the perspective of AT?
2. How does school EFL teachers’ research identity evolve within the U-S community?
3. What are the influencing factors in school EFL teachers’ research identity construction?

## Materials and Methods

### Context: U-S Community in China’s EFL Curriculum Reform Projects

The *English Curriculum Standards for Senior High Schools* issued by China’s Ministry of Education (MOE China, 2020) states that English curricula consist of compulsory, selective-compulsory, and elective courses. Among the three, the elective part includes school-based courses such as basic courses, practical courses, expansion courses, and second foreign language courses, aiming to foster students’ language competence instead of basic language skills. Furthermore, with the positive advancement of the new curriculum reform, many schools have started to work with universities to run school-based courses under the local government’s support. In this context, a community between the university and the school is formed.

In this study, three collaborative projects between the university and the school aim to complete the school-based course design under the curriculum reform. The three projects involve at least 12 EFL teachers from three schools and 12 EFL teachers from a key university of the “211 project” in Shanghai, China. All teachers agreed to participate in the community with the approval of their workplace. In the beginning, three teachers were invited and were chosen to be the new course teachers from three schools. Permission of the principal of each school to carry out the project was requested, and the teachers were informed beforehand of the project so that they voluntarily participated in the activities throughout the community. To better pedagogical practice, a range of activities in the community was arranged as follows: observation and evaluation of the class, workshops, and lectures, and achievement report communications. Apparently, the English curriculum reform is at the heart of this community; however, the research has influenced the teaching practice greatly and deserves more attention. In the U-S community, school teachers and university teachers often come together virtually and physically to fulfill the joint responsibilities of school practice and research. Therefore, school teachers’ research identity could shape and be shaped with the mentorship of university teachers.

### Participants

The middle school EFL teachers who had some academic achievements in the curriculum reform were purposely selected to explore the development of their research identities. Invitations to participate in this study were sent to the above teachers involved in the reform. The invitation explained that their participation would only concern the collection or analysis of data. Three middle school EFL teachers from three teaching projects in the U-S community consented to be the research participants for the present study. Each community appointed at least one university teacher to work with school teachers from each grade. In this regard, the university teachers who had advocated the construction of the school teachers’ research identity in each community are the focus of this study. Invitations were also sent to the university teachers, and three university teachers consented to participate in the present study. In summary, six teachers voluntarily participated in the research without receiving any type of compensation. All three university teachers (named UT1 to UT3) have experience cooperating with school teachers for more than one teaching year. All three female middle school teachers (named T1 to T3) hold master’s degrees and have joined the community for at least 1 year. Additionally, one university expert (named UT4) responsible for all three projects is also the focus of the study. The profile of the school teachers involved in this study is shown in Table 1.

**TABLE 1:** The profile of school teachers.

Teachers*	Gender	Age	Education	Title class	Years of teaching	Years of participation	Mentor
T1	Female	28	Master	Level 1	2	2	UT1/UT4
T2	Female	37	Master	Level 2	11	1	UT2/UT4
T3	Female	54	Master	Senior	32	2	UT3/UT4

*Pseudonyms. Profile collected in 2021 autumn term.

### Data Collection and Analysis

This case study adopts narrative case study methods within the qualitative research paradigm (Rushton, 2001) to address the research questions. Narrative inquiry tends to capture the story characteristics of human experiences and presents the research results in the form of stories (Clandinin and Connelly, 2000). Taking the narrative frame in the study of Barkhuizen and Wette (2008), who centered on the experience of Chinese foreign language teachers, and that of Gu et al. (2013), who emphasized EFL teachers’ research context, this study designed the frame based on the teachers’ profile, research history, reform history, research status, opinions on research requirements, research interests, and difficulties and expectations, to get information from three school teachers. When using narratives, the reliability of narrative text lies not in the statistical results but in the reliability of data analysis, and its validity aims not to explain authenticity but to prove possibility (Polkinghorne, 1988: 175–176).

To solve the problem of key omissions and ambiguities in narrative texts, especially to present more complete records, this study conducted a semi-structured interview with teachers either face-to-face or via WeChat. Interviews were conducted with school teachers and university teachers to investigate school EFL teachers’ research identities from the dual perspectives of participants on both sides in the U-S community. Questions in these interviews prompted teachers to recall the development of the U-S community and the influence related to the research within the community. For example, the participants were asked to talk about the reasons for their participation, the activity in the community, and the exchanges among teachers in terms of research. The questions were not given to them beforehand. The interviewer was a Ph.D. candidate who had established a friendly atmosphere while interviewing. During the interview, the interviewees were told that there were no right or wrong answers, and they could use stories to support their views. Each interview lasted about 50–90 min (507 min in total), and the interview contents were recorded and transcribed. Additionally, documents such as the meeting minutes were analyzed to probe into the research potential of school teachers in routine teaching practice. All data were collected in Chinese to identify the participants’ views accurately.

The purpose of the study was to describe the U-S community of Chinese curriculum reform and explore EFL teachers’ identity construction within the community. Therefore, the analysis focuses on the U-S community that EFL teachers described and the process and factors that influenced EFL teachers’ identity in the narratives and interviews. The analysis protocol contained a U-S activity system category involving subject, object, community, tool, rules, division of labor, and outcome (Engeström, 1999). Also, the analysis of conceptualized identity involved five essential activities: textual production, engaged projects, conference participation, academic visits, and higher academic degrees (Flowerdew and Wang, 2015; Zacher et al., 2019). Based on the literature mentioned above, data collected were coded to analyze participants’ identity development in NVivo 12. Two raters, including the first author and the other, who are both experts in qualitative research, coded from a total of 4,321 words of narrative texts and 131,155 words of interview texts. Before the work, raters initially coded 20 node references from the randomly selected texts without discussion. After achieving a consensus for each coding criteria, the raters completed the rest of the coding.

## Findings

### The U-S Community in the Project of EFL Curriculum Reform

**FIGURE 1:**
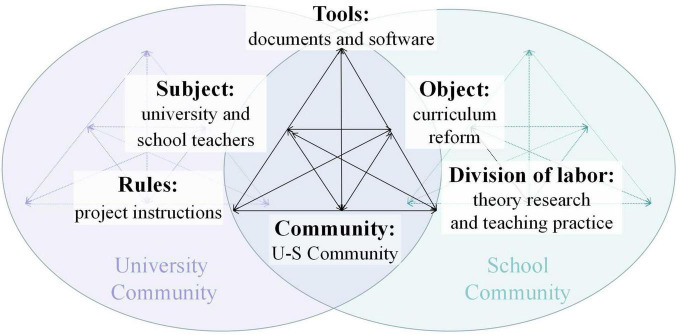
The U-S community in the Chinese EFL curriculum reform. [Adapted with permission of Cambridge University Press through PLS Clear from He and Lin (2013)].

The emergent community surrounding a university or school teacher has precipitated changes to their existing communities and a joint activity system that is boundary-crossing in the existing communities has been constituted (He and Lin, 2013). In this study, we call this shared system the U-S community which is overlapped for the shared community object in both the university community and the school community, as shown in Figure 1. In so doing, we show each element involved in the U-S community, and its mediational role in the three EFL in-service teachers’ research identity construction.

#### Subject and Division of Labor

University and school teachers are the backbones of the U-S community in the project of EFL curriculum reform. Most of them participate actively to promote their professional development; however, a small part of them join in by chance and consider the reform as extra labor, especially at the initial stage of the project.

Respecting the division of labor, the university expert team is responsible for the theoretical direction of the reform aim, philosophy, method, and resources, and the school practice teacher/team applies those ideas to practice. Different expert groups in the U-S community are divided by diverse research interests. As mentioned in the meeting minutes, university teachers interested in language teaching form the front-line expert groups and frequently communicate with front-line school teachers. Furthermore, the school teachers in the project have access to the corresponding teaching practice, reports, lectures, or workshops concerning teaching and research, which has improved school teachers’ comprehension of the theory and facilitated their identity change toward that of a “researcher.”

**Extract 1**

*The expert team who are interested in English teaching come to the school to guide the curriculum reform theoretically and conceptually, and provide more resources related to the teaching design in terms of the school teachers’ teaching practice. And the university experts from the field of applied linguistics are invited to deliver lectures or hold workshops on teaching and research (T2/UT2, 09-21-03*, meeting minutes).^1^

#### Tools and Rules

The tools and rules that school teachers perceive contain the documents and software. School teachers could find books and journals in the school library or the teaching and researching section. Other documents concerning curriculum standards and project-based instructions are the tools as well. Moreover, some online surveys are employed to probe into the needs or status of teaching and learning. Teachers may search for some data on the internet about teaching and researching, such as the China National Knowledge Infrastructure. All those documents consolidate the school teachers’ theoretical foundation and provide examples for research. By observing the teachers’ completed and ongoing research, it is evident that each chosen data collection and analysis tool is employed to answer the question that the teacher has proposed. For instance, online surveys are assigned to inquire about the needs or status of teaching and learning. The recording and transcription software is selected to collect the qualitative data (i.e., interview and classroom observation) to supplement the questionnaire data.

Considering the rules, school teachers generally mention the project instructions. However, there is no specific written rule but a rough instructional plan at the initial stage. Although different projects have different rules because of different objectives, the primary references are curriculum standards, project-based learning concepts, and English teaching principles. As shown in Extract 2, UT1 stated that they did not have definite and detailed regulations concerning the project when it first began. However, they gradually summarized the practice by observing the class and the discussion on the reform philosophy. As a result, a basic logical arrangement was formed and polished, such as the appropriate teaching design, principles, and methods.

**Extract 2**

*We didn’t have the definite rules at first [.] We gave them (school teachers) the general directions according to the reform concept and the philosophy of English teaching. Therefore, in the first year, we constantly refined and polished [.] By means of observing and evaluating the class, and discussing the reform concept, it has become clearer to us what goals we design, what principles and methods we use to achieve our goals, what activities we organize, and what processes we arrange (UT1, 12-21-22, interview).*

#### Community

There are comprehensive and detailed discussions between the university and school teachers regardless of the various project objectives in different communities. For example, T2 said she communicated with university teachers actively because she was the first one who took part in her school’s curriculum reform. Therefore, the U-S community became the window for T2’s exchange in teaching and research, and T2 had more frequent communication with the university teachers, which shows the U-S community has a paramount influence on T2’s research identity construction. In each community, the university teachers usually observe school teachers’ teaching/research and give their feedback. The school teachers typically state their reflections about what has been done in this class or this project. However, in terms of the content of the discussion, some teachers (e.g., T3 in Extract 4) pointed out that there still exist some problems, and the discussion would be more effective if there were more specific guidelines oriented directly toward the school teachers’ needs. In addition, the U-S community can invite experts from the university to deepen the discussion to help school teachers improve their teaching and academic research ability, as proposed in all three school teachers’ interviews. Furthermore, school teachers also stated that they want to send their research works to the university teachers for review. It shows the importance of the targeted discussion in the U-S community.

**Extract 3**

*Because I was the first one to participate in the reform and taught the course alone for one year, it seems that I talked a lot with the expert team about teaching and research. The experts help me check the theoretical framework and research methodology in my research process (T2, 01-22-29, narratives).*

**Extract 4**

*The teachers in our school do have a lot of work pressure. We are working against the clock on our work. So we may think communication is a waste of time if we don’t get specific help from the university teachers. Thus, it may be more effective if we have a candid exchange of ideas. We (school teachers) can raise our questions, and university teachers can give us more targeted support (T3, 01-22-07, interview).*

#### Object

Although the expansion course in each curriculum reform has its focus, the main aim is to expand students’ knowledge, promote teachers’ sustainable development, and improve teaching effectiveness. For a better teaching effect, teachers have spent a lot of time broadening their theoretical knowledge. Therefore, the theoretical exploration served as an important element in improving the teaching practice, and the theory adopted by the teacher was thoroughly tested. In doing so, school teachers’ teaching ability could be improved through the teaching practice, as T1 mentioned in the interview. Additionally, the school teachers’ research quality could also be enhanced; their research identities’ construction is shaped by the academic interactions with university teachers, as shown in T2’s interview.

**Extract 5**

*Teachers’ abilities in all aspects have been improved because it is a new course and a new project. We constantly discuss the curriculum design, the collection of materials, and the teaching process, which has improved our teaching abilities (T1, 01-22-27, interview).*

**Extract 6**

*In terms of the research, UT2 and UT4 will communicate with me regularly. This kind of communication encourages me to write more papers, do more research, and learn more, then I have got some achievements (T2, 01-22-23, interview).*

### School EFL Teachers’ Research Identity Construction in the U-S Community

The school-university collaborative program helps form a U-S community, which could influence school EFL teachers’ research identity. To be specific, school EFL teachers might experience identity construction in a highlighted spiral of “practitioner” and “researcher” in this activity system.

### The U-S Community Is a Melting Pot of Teaching, Research, and Life

Although all three projects are concerned with curriculum reform, all three school teachers’ professional development concerning research is enhanced. By participating in the reform project, three teachers get access to the activities of writing research papers, applying for research projects, and attending academic conferences. However, along with the implementation of the curriculum reform, both the school community and the university community inspire school teachers to increase academic output with maximum effort. The headmaster and project leader encourage school teachers to have scholarly output in the school community. As T1 mentioned:

**Extract 7**

*The headmaster and leader of the project team said that they hope we (school teachers) could make some achievements in conducting this project (T1, 01-22-27, interview).*

In the university community, university teachers would like to discuss the research issues and methods with school teachers and invite professors or journal editors with expertise in English education to examine school teachers’ manuscripts or give lectures. For example, UT3 and UT4 shared their experiences in Extracts 8 and 9.

**Extract 8**

*In fact, we cooperated through this project with T3 [.] I would listen to the teacher in class and then participate in their teaching and research activities [.] I helped her revise the manuscript by offering some advice [.] And after writing it, she felt she needed to polish it. At this time, she would invite a university teacher to give her some suggestions (UT3, 01-22-06, interview).*

**Extract 9**

*During the project, we periodically invite well-known experts to give some lectures [.] aiming at improving the school teachers’ academic competence [.] we hope they can write some related articles and then guide them in writing or give some recommendations for the selection of target journals (UT4, 01-22-04, interview).*

On the other hand, school teachers may have academic research needs, wishing the university teachers to help them through teaching cooperation and research guidance. As T3 shared,

**Extract 10**

*During this project, we found that our teachers’ teaching philosophy also needed to be further improved. We thought that academic paper writing was a very good way. And we also needed papers to meet the professional title requirement [.] The teachers in the program hope to get help in writing papers from the university (T3, 01-22-07, interview).*

As for the contents of guidance, T2 described the kind of support that school teachers need to get while doing research:

**Extract 11**

*For example, if we (school) teachers have some questions or puzzles in teaching, and we want to make further study, the university teachers could help us transform the questions into a research direction or topic. We also want to know how to further find some relevant literature and some papers to study, and then how to construct the framework of the whole paper, including further refinement, even the modification of the language (T2, 01-22-23, interview).*

Interestingly, besides teaching and research, the U-S community also encourages emotional communication of teachers in daily life to make teachers happier and relaxed. As T2 shared,

**Extract 12**

*Sometimes, in addition to talking about research and teaching, maybe I also talk about issues in life with UT2 because I am more familiar with her [.] In fact, talking about life is also a part of self-development, because as teachers, no matter you are teaching, working, or doing research, you are actually supported by your own active attitude toward life and wisdom [.] When we are going to have formal discussions, we definitely focus on some teaching and research aspects, but I am also trying to build a closer connection in life outside of the work (T2, 01-22-23, interview).*

### School Teacher Identity Transformation: A Spiral Process

EFL teachers’ identity develops from being a practitioner of ordinary teaching to being a researcher with the start of the curriculum reform. As the project moves on, their identity transforms from being a researcher to being a practitioner to validate the research findings gained from teaching. In the U-S community, the university teachers think that school teachers can receive their guidance with greater enthusiasm if they help school teachers do research. As UT1 mentioned his reasons for encouraging school teachers to do research:

**Extract 13**

*The reason why I encouraged school teachers to do research was that I wanted to make them more able to accept the things we spontaneously do for them from an emotional perspective other than guiding class directly. Because I felt that their participation would be passive when theories were ambiguous to them. By writing research papers, school teachers may improve their research skills to a certain extent or participate in the course with more enthusiasm (UT1, 12-21-22, interview).*

Through participating in the reform and the writing related to the reform, school teachers’ identities gradually transformed from practitioners into researchers. From the narratives, all three school teachers have shown a strong record in academic output focusing on curriculum reform. Concerning how school teachers felt about the transformation from practitioner to researcher, T2 mentioned that the research ability or academic awareness could be improved to a certain extent. For example, she would observe others and then make some comments. By looking back on these, she found that it also had some influence on herself.

**Extract 14**

*Well, by observing other teachers’ teaching, I found that I have got the skills of assessing a class instead of merely teaching from experience, which means I know whether the teaching is good or not. After doing certain research, I not only understand whether the class is good or not, but I can also tell why it is not good, and how to make it better. I can give targeted suggestion based on my research, which means I know the causes of certain teaching problems and what teaching strategies are more appropriate (T2, 01-22-23, interview).*

Significantly, after transforming from a teacher to a researcher, there will be the transformation from researcher to a teacher again. The procedure is spiral. Regarding the relationship between teaching and research, T2 pointed out that it is similar to the relation between theory and practice, which could not be separated and go both ways.

**Extract 15**

*I think the relationship between teaching and research is like that between theory and practice. My teaching is a practice, and then research is the theory which guides the teaching [.] However, if you have done certain research, and you have got a guiding principle and strategy, it is certainly better for you to practice. But after practicing for a while, you may be a little confused, so you’d better go back and review your theory again. After the research, in fact, I think you still need to make a second leap in the end and return to practice. Otherwise, it’s all just empty talk (T2, 01-22-23, interview).*

The seasoned academic T3’s practice for years also supported this opinion of T2. T3 mentioned that she would like to find the solutions to teaching problems by searching, researching, and applying the ideas to teach to verify whether the hypothesis is appropriate. Then, after practicing, some conclusions and reflections may be drawn for the academic output, which forms a circle.

**Extract 16**

*When you encounter problems in teaching, you have to think about how to solve them. Then when you are looking for a solution, you will inevitably search for literature, audit your colleagues’ classes, and sit in lectures by experts attentively. In this process, you want to solve the problems on your own. This kind of thinking will gradually become clear. Then in the second year of teaching, you can put the idea into classroom teaching to verify whether it is practical. That is to say, during this process, if you record your thoughts, reflections, and achievements in time, in the end, your academic output will naturally fall into place (T3, 01-22-07, interview).*

### The Influencing Factors in School EFL Teachers’ Research Identity Construction

School EFL teachers’ identity construction is influenced by internal factors, including research experience and stage of teachers’ career, and external factors such as the curriculum reform opportunity and the way to communicate in the U-S community. For instance, in the narratives, T1, who now is the Level-2 teacher,^2^ mentioned that she received academic research training by doing her master’s thesis 2 years ago. After becoming a school teacher, she successfully applied for and completed the district-level project in Shanghai. She was also writing a paper relating directly to the course. In the interview, she explained there was relevance between her research direction and the course in the reform, which indicates the continuity of her research interest, showing a connection between her study and work, which typically appeared in the anticipatory career stage (Steffy, 1989).

**Extract 17**

*I graduated in 2019, majoring in foreign linguistics and applied linguistics. I am the second class teacher in the school now. There is no research requirement for professional titles of this level [.] Since joining the school, I have successfully applied for one district-level project, and completed it [.] I am also writing a paper on curriculum reform (T1, 01-22-25, narratives).*

**Extract 18**

*I have published a paper with my supervisor during my master’s study [.] I further explored this research scope in the project [.] I take this course, which is my integration course, as one case for my research project (T1, 01-22-27, interview).*

Although T2 had not written any papers until she joined the curriculum reform, she had written three papers by the time of the interview. She had confronted some challenges during the writing, especially in the organization of the paper, because she had nearly forgotten how to write a paper. However, gradually, with the help of UT2 and UT4, she refreshed herself in writing papers again.

**Extract 19**

*It is the first time that I have completed a paper related to the research topic of my master’s thesis since I graduated in 2010. Because I don’t need professional promotion urgently, I haven’t taken writing papers seriously [.] After completing this course, I am thinking about writing a paper on teaching [.] However, in the beginning, the ideas are scattered. I have written a lot, but there isn’t any focus [.] there isn’t a structure, but gradually, after communicating with UT2 and UT4, I kind of recalled how I wrote my paper when I was studying for a master’s degree (T2, 01-22-23, interview).*

Unlike TI and T2, T3 was awarded a senior professional title in 2001. Over the past 20 years, with enthusiasm for research, T3 has continued to do research and wants to pass on her experience to others. In addition, she might prepare to enter the exit stage of career development (Steffy, 1989). She mentioned that she did not need any research help because she would retire the following year. She seemed to pursue another identity as a teacher educator because she said she had organized district teachers to do research.

**Extract 20**

*I was a senior class teacher in 2001 [.] I gradually formed a habit of reflecting on my teaching[.] In fact, I am not so eager to get any help on research now because I’m retiring next year [.] This year, I want to write some of my experience in a paper. Maybe I’ll finish it next year [.] Last year, I cooperated with another teacher to set up a backbone teacher workshop in the Jing’an District of Shanghai [.] There were a total of 13 teachers in our workshop [.] They wrote papers on their teaching [.]In this process, it was actually a kind of cultivation of their research awareness (T3, 01-22-07, interview).*

Aside from the above-mentioned internal factors, curriculum reform is an opportunity for teachers’ improvement and growth. As T2 said, the curriculum reform could provide novel teaching and research topics, leading to gradual changes in traditional teaching and research.

**Extract 21**

*I didn’t seem to be very active in taking this task at first [.] I did consider it as a way to get rid of such a sense of burnout in daily teaching [.] that is, by the 10th year of teaching, I think that teaching without any new method or role is less attractive [.] So, when the workload is reduced, on the one hand, it is more comfortable, but on the other hand, the feeling of more comfort does not mean achievement [.] I do hope to make more breakthroughs to do some meaningful things concerning various aspects, including the design of a new course, the participation in the teaching competition or writing some research papers (T2, 01-22-23, interview).*

Also, in the context of curriculum reform, teachers’ research practice may be influenced by a strong positive atmosphere of research. For example, all three teachers in the reform team of T1’s school did some research, inspiring and encouraging her to do research.

**Extract 22**

*The teachers consider it as a very good opportunity to participate in this project. They can suddenly have a new topic, or a new research direction, and can make their own achievements [.] all three of us in the project do both teaching and research, (that is) 100% [.] The atmosphere of research will also influence me, because I think during this process we can communicate with each other and inspire each other, and then we can encourage each other. In fact, this atmosphere is very positive and powerful (T1, 01-22-27, interview).*

Regarding the external factors, communication in the U-S community is another element that influences the school teachers’ identity construction. University teachers have all mentioned that their guidance to school teachers was only an external factor, reducing the pressure on both parties and relaxing the atmosphere. For example, UT1 mentioned that the guidance from outside is not decisive, but the individual school teachers play a decisive role. UT2 also mentioned that if she encouraged teachers too deliberately, it might bring about the complaints/grunts of school teachers, which is not conducive to establishing harmonious relationships. Thus, it is recommended to integrate the object of doing research with discussions on teaching practice to reduce the research pressure on the school teachers. As a result, a relaxing atmosphere subtly influences teachers’ identity construction from “teacher” to “researcher” when research pressure is reduced by communication.

**Extract 23**

*First of all, I think the teacher still needs to have this kind of awareness because he or she can be stimulated only by themselves (in doing research). If he or she has this awareness and regards it (doing research) as a good opportunity, he or she can discuss it together. In other words, they need inner drive. For me, I can give some opinions and suggestions, but they are not enough to motivate them (UT1, 12-21-22, interview).*

**Extract 24**

*Well, the encouragement is sometimes related to our positioning. For example, when I give suggestions, she (the school teacher) may wonder why I am in the position to do it. In fact, I would like to be available and supportive at any time when the school teacher has such research needs. If the teacher doesn’t need my suggestions, I think maybe I’m not in the position to persuade them or to guide them. Please don’t misunderstand, it doesn’t mean I am not ready to help. As far as I know, some school teachers seemed not so eager for doing research; they even disliked more reforms (UT2, 01-22-30, interview).*

However, when the university teachers’ responses to school teachers’ needs are different, the guidance effects vary accordingly. For example, when T1 was preparing her project, UT1 recommended and shared some related books via WeChat. However, there was not much further communication on research into the project. T1 did not mention some discussions with UT1 concerning her research paper, which seemed that the communication between them only concerned recommending relevant literature. However, unlike TI and UT1, T2 pointed out she had more frequent and timely discussions with the university teacher virtually, which immensely supported her research.

**Extract 25**

*In terms of the research, when I was doing a project, UT1 gave me some relevant books via WeChat [.] I think it was helpful because the materials were related to my research direction when I was a graduate student. So I think the materials are very helpful theoretically (T1, 01-22-27, interview).*

**Extract 26**

*After completing a project last year, UT4 urged me to write three articles so far [.] We keep in contact at least once a month [.] We use WeChat to communicate[.] The shortest time is about 10 minutes [.] The longest may be an hour or so (T2, 01-22-23, interview).*

According to the meeting minutes, T2 had discussed the targeted journals, writing styles, and comments from the journal editors with the university teachers. From the content, the problems in the research have been closely examined. With mutual communication, on the one hand, the university teacher could know more about the school teacher’s needs and offer timely, personalized, and targeted support, as UT2 said in Extract 27. On the other hand, the school teacher could get more trust from the university teachers and feel more comfortable and confident in identity development in the U-S community, as shown in the experience of T2 in Extract 28.

**Extract 27**

*I think interpersonal communication is like a process of interaction and mutual attraction. Well, on the basis of doing things, we communicate more, which sometimes forms a friendly interaction, right? Well, if you communicate more, you will know more about her(the school teacher) needs, so this kind of support will be more targeted (UT2, 01-22-30, interview).*

**Extract 28**

*I trust our team a lot [.] There is someone who helps me (in my teaching and research), and we learn together. I trust you (university experts) very much, and then I know I will become better with your help and have better self-development [.] You are doing things seriously and rigorously [.] The communication is also very efficient. If I need any help or support, there will be a response as soon as I tell you. It is always timely and helpful (T2, 01-22-23, interview).*

## Discussion

### Interpretation

#### The U-S Community in the Project of EFL Curriculum Reform

This study stems from recent studies that focused on the partnership between the university and the school and showed that the U-S community has some influence on teachers’ identity development (He and Lin, 2013; Yuan and Burns, 2017). In many cases, teachers constructed their identity in the U-S collaborative project-oriented to professional development (Yuan and Burns, 2017; Kenan and Demet, 2018), ignoring the focus of the U-S collaborative project for curriculum reform. The present research explores whether school EFL teachers’ identity is associated with the U-S community in the project of EFL curriculum reform. Coupled with the study of He and Lin (2013) that reported on teachers’ identity construction in the reform context, the present study’s findings show that the U-S collaboration program helps form a U-S community, which influences school EFL teachers’ research identity construction. The encouragement and needs are visible all over the U-S community, especially in cultivating teachers’ awareness and ability to do research. Furthermore, this study finds that the teaching-oriented community can also influence teachers’ research identity. Concurrently, communication promotes the harmony of the community and the development of teachers’ identities.

#### School Teachers’ Research Identity Transformation Process

**FIGURE 2:**
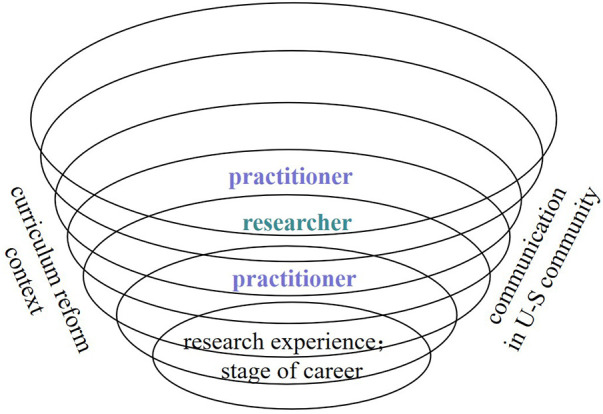
School teachers’ research identity transformation and its influencing factors.

The study investigated school EFL teachers’ research identity construction. The idea of a research-oriented U-S community (Yuan and Burns, 2017) is not innovative, and the reform-based U-S community is seldom treated as a context for teachers’ development (He and Lin, 2013; Han, 2021), whereas few studies reflect the ongoing research identity construction. Akin to EFL teachers’ identity construction from practitioner to researcher (Goodnough, 2010; Schutz and Hoffman, 2017: 9; Yuan and Burns, 2017), our findings related to school teachers’ research identity formation portray a clear trend of teachers’ becoming researchers (as shown in Figure 2). It seems that school teachers gradually understand the philosophy of the reform and realize the importance and benefits of participating in a reform. To an extent, the profound understanding has stimulated school teachers to accept the guidance from university experts voluntarily, and this deep participation in the curriculum reform guides school teachers to carefully reflect on the lessons learned, which promotes the process of being a researcher (Allwright, 2003; Liu et al., 2021). Moreover, differing from previous studies, our findings also sketch a spiral route of teachers’ identity formation shifting from researcher to practitioner (as presented in Figure 2). It can be argued that the school teacher’s research identity, constructed from doing research, will finally be embodied in more targeted teaching practices (Zhuang and Qi, 2015). In alignment with this perspective, school EFL teachers’ identity constructs a spiral process that improves both teaching and research.

#### The Influencing Factors in School EFL Teachers’ Research Identity Construction

This study identifies the influencing factors in school EFL teachers’ identity construction through multiple perspectives by analyzing interviews, narratives, and other documents. Consistent with the analysis of both internal and external factors (Peker et al., 2020), the present study shows that EFL teachers’ identity has developed mainly around its internal factors of school teachers’ research experience and stage of teachers’ career, and external factors concerning the curriculum reform context and the communication in the U-S community (as depicted in Figure 2). In line with the relevant studies (He and Lin, 2013; Aderet-German et al., 2019; Jiang, 2022), as the research shows, school teachers resort to research when they regard the reform as an opportunity for self-development and when they feel secure and relaxed in the U-S community. Additionally, communication in a relaxing atmosphere can reduce the research pressure on both parties by integrating the research into daily teaching practices, which subtly influences teachers’ identity formation from being a “teacher” to becoming a “researcher.” Likewise, the virtual communication between the university and school teachers through WeChat or email facilitates university teachers to understand school teachers’ needs and provide targeted content in both daily and formal interactions frequently, therefore reducing tensions or conflicts (He and Lin, 2013; Yan and Yang, 2017) in the community, improving mutual trust in their cooperation, and building harmonious relationships in the community (Li and Liu, 2021). Moreover, this study claims that school teachers’ identity construction regarding research has a much closer relationship with individual experiences, such as personal research experience and stage of teachers’ career (Goodnough, 2010; Livingston, 2016), and implies the importance of improving teachers’ research ability in order to foster teachers’ research identity.

#### Limitations

With a few findings on school EFL teachers’ research identity, the study has several limitations. First, it only explores the identity development of three school EFL teachers with academic output from participating in the curriculum reform of a U-S community. Future studies may utilize different research methods, especially quantitative methods, to examine school EFL teachers’ identity development in U-S communities. Second, since there are disparities in three U-S communities among cooperative projects, how school EFL teachers’ identity reacts to such differences is not fully explored. Additionally, the present study only investigates school teachers’ identity in the U-S community; more perspectives can be explored in the future.

#### Implications

The findings of this study have several implications for school EFL teachers, university teachers, and policymakers. First, it recommends that school teachers actively and positively participate in the project of EFL curriculum reform in Chinese basic English language education because the reform endows teachers the alternative possibilities to develop their identities (Aderet-German et al., 2019; Jiang, 2022). Echoing the implications of similar research (He and Lin, 2013; Jiang, 2022), school teachers may experience a new identity by moving out of their previous comfort zone. Moreover, school teachers could get the opportunity to communicate with and learn from the university teachers on relevant theories in the U-S community, which consequently influences the development of identity for research to some extent. Second, university teachers, on the one hand, could encourage school teachers to do action research (Burns, 2009; Pennington, 2015) or exploratory practice (Hanks, 2014, 2019) by sharing some reference materials or soliciting notice for conferences, journals, or projects at home and abroad from the perspective of researchers. On the other hand, university teachers could more actively communicate with school teachers by offering timely and targeted responses to the school teacher’s needs. In doing so, an effective and trustworthy interaction can be established. Finally, the policymakers responsible for the teaching plan and school management could continuously refine the objects and division of labor in the U-S community (Yuan and Burns, 2017). Even if the community mainly aims to improve teaching practice, it can also shed light on improving teachers’ research ability and increasing teachers’ practice-oriented research output. Accordingly, arrangements for this object are presented in detail because only the maximal cooperation in labor division for the same object can accurately supply the research resources and mobilize these resources to meet school teachers’ needs to ensure the maximum reasonable utilization of different activity systems in the U-S community (Engeström, 1999). For example, the university community could provide more experts to give academic lectures, review manuscripts, etc. Furthermore, the school community could call for participants and shoulder the responsibilities of investigating the teachers’ needs and offering managerial support.

## Conclusion

This study explored the identity construction of three school EFL teachers engaged in their respective U-S communities. The study focuses on teachers’ research identity construction by resorting to activity theory. The findings have concluded that there was a U-S community along with the collaborative project of the university and the school, and school teachers’ research identity was constructed by the community, forming a spiral cycle from the practitioner to the researcher, under the impact from both the internal factors containing the research experience and the stages of teachers’ career and external factors such as the curriculum reform context as well as the communication in the U-S community.

## Data Availability Statement

The original contributions presented in the study are included in the article/supplementary material, further inquiries can be directed to the corresponding author.

## Author Contributions

Both authors listed have made a substantial, direct, and intellectual contribution to the work, and approved it for publication.

## Conflict of Interest

The authors declare that the research was conducted in the absence of any commercial or financial relationships that could be construed as a potential conflict of interest.

## Publisher’s Note

All claims expressed in this article are solely those of the authors and do not necessarily represent those of their affiliated organizations, or those of the publisher, the editors and the reviewers. Any product that may be evaluated in this article, or claim that may be made by its manufacturer, is not guaranteed or endorsed by the publisher.

## Footnotes

1 In (T2/UT2, 09-21-03, meeting minutes), T2 and UT2 refer to the subject, 09-21-03 represents the date, September 03, 2021, and meeting minutes symbolize the text source.

2 The titles of middle school teachers are divided into five grades from the senior title to the staff level, which are high senior, senior, first class, second class, and third class teachers.
